# Study Protocol for Evaluation of an Extended Maintenance Intervention on Life Satisfaction and BMI Among 7–14-Year-Old Children Following a Stay at a Residential Health Camp in Denmark

**DOI:** 10.3389/fpubh.2021.733144

**Published:** 2021-11-23

**Authors:** Mette Juul Kristoffersen, Susan Ishøy Michelsen, Mette Rasmussen, Pernille Due, Lau Caspar Thygesen, Rikke Fredenslund Krølner

**Affiliations:** National Institute of Public Health, University of Southern Denmark, Copenhagen, Denmark

**Keywords:** study protocol, practice-based research, long-term effects, maintenance intervention, residential health camp, children, life satisfaction, overweight and obesity

## Abstract

**Background:** It is challenging to maintain effects of public health interventions. For residential health camps benefits often disappear as the child returns home. Furthermore, long-term effects are often not measured or reported. This paper presents the study protocol for an evaluation of an extended maintenance intervention offered to children who have completed a 10-week residential health camp at one of the five Danish Christmas Seal Houses (DCSH). The target group of DSCH is 7–14-year-olds with social, mental, and/or overweight issues and the overall aim of the camp is to increase life satisfaction and a healthy lifestyle. The primary aim of this study is to assess the effectiveness of the maintenance intervention on children's life satisfaction (primary outcome) and BMI *Z*-score (secondary outcome) 1 year after health camp.

**Methods:** The extended maintenance intervention is developed by DCSH and delivered to each child and family individually by an intervention coordinator to help children maintain positive benefits of the health camp on life satisfaction and health behaviors after returning to their homes. Intervention activities target the child and the family. The effect will be tested in a quasi-experimental design: The intervention is offered to half of the children at one of the five DSCH (intervention group, *N*~144) while the other half and the children at the other four DSCH receive a standard maintenance intervention (control group, *N*~894). Children will complete questionnaires on life satisfaction measured by an adapted version of the Cantril ladder and height and weight prior to health camp, at the end of health camp, 3 months and 1 year after the end of health camp. To enable per protocol analysis and nuanced interpretation of effect estimates, we will monitor the implementation of the intervention by a process evaluation study among children, parents, and follow up coordinators using qualitative and quantitative methods.

**Discussion:** We present a systematic approach to evaluating practice-based interventions in a research design. The study will provide new knowledge on the effectiveness of individualized maintenance interventions on long-term effects on life satisfaction and weight loss among children.

**Trial registration:** Prospectively registered at Current Controlled Trials ISRCTN 13011465 https://www.isrctn.com/ISRCTN13011465

## Introduction

Low life satisfaction and overweight are public health concerns among children and adolescents in Denmark as well as in other western countries (1, 2). Overweight and indicators of mental health and subjective well-being (life satisfaction, health-related quality of life) often co-exist i.e., children with overweight often also struggle with physical, mental and social health issues (3, 4), which have a negative impact on their quality of life (5) and with life satisfaction (6). This co-existence has been addressed in several public health interventions. Results on intervention effects on well-being and maintenance of weight loss are limited due to insufficient evidence, however, a review from 2014 found a trend toward effect on quality of life in multidisciplinary interventions (7).

In Denmark, 6–11% of 11–15 year-old children indicate low levels of life satisfaction (8) and 14% of children live with overweight when entering primary schools (9). Both poor life satisfaction and overweight have great impacts on everyday life in childhood and adolescence and are often strong predictors for facing similar problems in adulthood with mental health problems and lifestyle diseases as main consequences (10, 11). Therefore, it is of great importance to apply appropriate interventions which address the broad psychological needs (e.g., low life satisfaction, negative body image, and social isolation), train skills and provide support for behavioral change (12).

A large meta-synthesis found a significant effect of obesity treatment interventions on children and adolescents, however with relatively little clinical relevance, since the changes in BMI *Z*-score (standardized body mass index score for specific populations) were small (13). Another study looked at maintenance of weight loss results after treatment of childhood obesity and found that BMI-*Z* score for the maintenance intervention group remained stable compared to a slight increase in BMI-*Z* score in the control group (14).

To address problems with overweight in 6–11-year-old children, there has been a greater focus on multi-behavioral (diet, physical activity, behavior-changing) interventions in the international literature (15). These interventions have only achieved small, short-term reductions in BMI thus there is a need for long-term follow up and further research to identify maintenance interventions to sustain weight reductions (15). Maintenance interventions are a programmed additional dose of intervention to sustain positive effects of the initial interventions (16). Parental involvement and interventions addressing the home environment may also be effective in reducing overweight among children (17).

Residential programs are sometimes used as an approach to address multiple health behaviors along with mental and social problems in children. Positive short-term effects of these programs on weight loss have been reported whereas reports on long-term effects are limited (18, 19), which is also the case for residential programs in Denmark such as the health camps called the Danish Christmas Seal Houses (DCSH) (20).

Thus, there is a need for developing and testing appropriate forms of maintenance intervention to ensure intervention benefits are sustained over time (14, 15).

For residential health camps, one major challenge for child participants is to maintain a healthy lifestyle when returning to their homes after the end of a stay. At the health camp, the staff and physical surroundings ensure a healthy life style and a focus on well-being, while the home setting often requires a change of behavior for the whole family in order to obtain a healthy lifestyle (21). To support the child in maintaining their weight loss and increased level of life satisfaction, a maintenance intervention should target both the child, the family, and the school and local community. The child must learn to resist unhealthy temptations in the home environment and maintain the positive belief in themselves which has been built op at the camp. Parents need to be role models and make healthy food and emotional support available at home. Schools and communities need to create environments, which promote emotional well-being and make healthy choices the easy choices. A study from 2007 did not find a long-term (1 or 2 years) effect on maintenance of weight loss of either a behavioral skills maintenance intervention or a social facilitation maintenance intervention, compared to a control group in children aged 7–12. However, for both maintenance interventions, a subset of children with low social problems, showed better long-term weight loss maintenance compared with children in the control group (22).

In Denmark, DCSH, offers a 10-week residential stay at one of their five health camps to children and adolescents aged 7–14 with social, mental, and/or overweight problems. The camps have existed in more than 100 years and used to have a special focus on secondary prevention of overweight. In recent years the main purpose of the health camp has been changed and it now aims to increase life satisfaction among participating children, through healthier lifestyle, more physical activity, supporting social relations, and improvement of individual competences such as self-esteem. To maintain the positive changes in the child after moving from the camp- to the family-setting, DSCH has developed two different maintenance interventions. A simple low cost 9-week maintenance intervention which is offered to all children staying at the five health camps (standard maintenance intervention) and a more comprehensive, and thereby resource demanding 16-week maintenance intervention which is offered to half of the children at one of the camps. Both interventions involve the whole family as a target group.

The core of the extended maintenance intervention is an intervention coordinator, who facilitates the transfer of knowledge, competences, and experiences from the camp. The coordinator aims at improving the collaboration among all stakeholders including e.g., parents and teachers in the home, school, and local environment of the child.

The idea is that key people in the child's home environment should help support the child in maintaining the weight loss and better life satisfaction achieved at the camp.

The purpose of this paper is to present the study protocol for the evaluation of the extended maintenance intervention of the DSCH camps compared to the standard maintenance intervention (control group). This includes the process and effect evaluation design, the intervention content, and the procedures for data collection.

The primary objective is to assess the effectiveness of the extended DSCH maintenance intervention at one of the health camps compared to the control group (standard maintenance at all five health camps) on life satisfaction in children 7–14 years old 1 year after the completion of a 10-week health camp (primary outcome). The primary hypothesis is that children receiving the extended maintenance intervention will maintain a higher life satisfaction after participating in the health camp compared to the control group.

The secondary objective is to assess the effectiveness of the extended maintenance intervention compared to the control group on self-reported BMI in children 7–14 years old 1 year after the completion of health camp (secondary outcome). We hypothesize that children receiving the extended maintenance intervention will be more likely to maintain a healthier BMI compared to the control group.

The following research questions form the basis of the evaluation:

Process evaluation:

- What are the characteristics of the children and families maintaining their results after 3 months and 1 year despite not receiving the extended maintenance intervention?
- What are facilitators and barriers in implementation of the extended maintenance intervention in the family and from intervention coordinators?
- What was the actual dose of intervention in the standard vs. the extended maintenance intervention and the association to the primary and secondary outcomes?

Effect evaluation:

- Can an extended maintenance intervention after participating in a residential health camp maintain results on the primary outcome life satisfaction measured on the Cantril ladder and secondary outcome BMI 1 year after the completion of health camp (primary and secondary outcome) or at short-term follow-up (3 months after health camp)?
- Can an extended maintenance intervention after the residential health camp maintain results on intermediate outcomes: self-efficacy, self-esteem, social relations, health promoting behavior (food, exercise, sleep), school satisfaction, body satisfaction, and parental health promoting behavior, and parental self-efficacy after 3 months and 1 year.

## Methods and Analysis

### Study Setting

#### The Danish Christmas Seal Houses and the Health Camps—A Historical View

In Denmark there is a national focus on childhood overweight and obesity with several initiatives and interventions carried out mainly by volunteers or in municipality settings (23).

Since 1912, DCSH, a private charity foundation in Denmark, has offered sick or vulnerable children a stay at one of their camps. In the early years, focus was on children with tuberculosis, asthma, or other diseases. In the 1960s focus changed to children with overweight and in recent years there has been an increased focus on children with low life satisfaction and social and mental problems. DCSH has thereby adopted the WHO's broad definition of health and includes both physical, social, and mental health in their definition of health among children and adolescents.

DCSH currently runs five health camps in different parts of Denmark. The camps are called Fjordmark, Hobro, Skælskør, Kildemose, and Liljeborg and they offer stays for children from all municipalities in Denmark. The five camps can accommodate between 24 and 48 children at a time with a total of 990 children staying at one of the five camps per year. The children stay at the camp for 10 weeks but return to their homes on the weekends.

The camp accepts children of the age of seven to 14 years (see referral to camp below). Most participating children are between 11 and 14 years old. The distribution of gender is approximately 50/50 with a small overweight of girls. Approximately 5% of the children have a different ethnic background than Danish compared to 11% in the general population. More children attending the health camps have physical or mental problems compared to other children of the same gender and age (24). Compared to other children of the same age, a larger percentage of children attending a health camp live together with parents who have no education or job and/or are divorced or live apart (25). The children struggle with different problems such as low self-esteem, bullying, and/or low life satisfaction. A recent study reveals that 97% of children at the health camps have either overweight or obesity problems (24). Prior to the camp stay, approximately 73% of the children report having a low quality of life (24).

The effectiveness of the camps has been investigated earlier, mostly in smaller studies. In 2008, a study of intervention activities at the Skælskør camp found significant weight reductions by the end of the program compared to baseline (20). An program evaluation of two of the health camps concluded that parents lacked support in order to be able to change their own lifestyle and to support their children in the process of returning home after their stay at camp (26). Historically, DCSH has focused solely on the child and on the time spent at health camp. The earlier vision was to let the children return to their homes with new confidence in themselves, having experienced a remarkable change of lifestyle and self-confidence.

The findings from 2008 were supported by another evaluation of a project which was conducted at two of the camps, Skælskør, and Hobro from 2013 to 2016, with the aim of improving the collaboration between the camps, the families, and home municipalities. The evaluation concluded that the children benefitted greatly from the health camp with higher quality of life, increased belief in themselves, less bullying in their school environment, feeling healthier and losing weight. However, it also highlighted that the children would be expected to benefit more from a more extended collaboration between the health camp and the parents, schools, and municipalities. Due to low response rate with only 5% answering all the questionnaires including the long-term questionnaire 1 year after camp the evaluation had serious limitations especially on the long-term effects 1 year after camp (27).

Based on the recommendations from this evaluation, the two different maintenance interventions included in this study as intervention- and control group have been developed.

#### The Content of the 10-Week Health Camp

The DCSH defines the goal of the health camps as increased life satisfaction among children. According to their program this goal is achieved through healthier diet, more physical exercise, better school performances and a pedagogical philosophy focusing on a strong community at the camps, and support of the development of the children (28). To accomplish these goals, the camps plan a structured and predictable everyday with various planned activities to support the children's self-development. To strengthen children's belief in themselves and to bring their experiences back to their home environment, the camps have a general philosophy and focus on changing the child's narrative e.g., from a ‘child who gets bullied' to ‘child who is good friend'.

When a child is accepted for a stay at one of the camps, the family is invited to an individual pre-visit ~2 months before the stay. During this, they are shown the facilities and are offered the opportunity to address any concerns and uncertainties. The children start at the health camp in groups, and the family is also invited to an information day along with other parents with children in the same group. This information day is scheduled to take place 2–4 weeks before the stay. The families are gathered to meet each other and for the children within the same group to get to know each other.

The 10-week stay at health camps is structured around sleep, meals, and different activities. The children get up and go to bed at specific hours, and in the morning they all go for a short run. Afterwards they go to school on site or attend other activities such as cooking classes, sport activities, talks about different aspect of well-being with reflections on issues like bullying, life satisfaction, how to treat others respectfully etc. After lunch the children have 1.5 h off where they can relax in their bedroom, play board games with others from their group, read, do homework or other quiet activities. In the afternoons and evenings, the children choose from different social activities such as painting, sports activities (e.g., ball games, spinning or fitness) and occasionally watch a movie or play computer games. When the children choose to participate in certain sports activities, they earn points in an internal competition. When a certain amount of points has been collected, they receive a sweatshirt. Often, the staff has organized a common activity for all children in the afternoon or evening.

The children can use their mobile device every day from 5 to 5.30 pm. At all other times it is locked away.

The children are not attending their own schools while at the camp. Instead they go to school at the camp in small groups of 6–12 students. They go to school for 12 lessons per week, which is a lot less than in their own school. They work with a lesson plan from their own schools and are taught Danish, mathematics and a foreign language. The children go to school for 1.5–3 h per day.

Three main meals and two healthy snacks are served at specific times each day and there are specific rules for maximum portion sizes allowed and requirements of eating a minimum amount of vegetables which is individually adjusted for each child. Everybody will at least taste the different components of a meal such as different vegetables. The meals served are healthy meals following the national guidelines for dietary advice in this age group (29). This also means that sugar and fat intake are limited but not prohibited. The philosophy is to give children knowledge about food and exercise by showing them how to cook, eat varied, and discover the fun of new ways to exercise.

#### Referral to Health Camp and Characteristics of Children

Any child and their family can apply for a stay at a DCSH health camp, and it is free of charge since DCSH raises money to run the camps. In the application form, the child, family, or other relevant people describe the rationale for why a stay at a health camp is thought to be a good idea for the child including the concerns and challenges the child faces. The application must be supported by the school and the child's family physician along with a social worker or a psychologist if applicable. The application is sent to DCSH, who distribute the applications to the principal at one of the five camps. The principal assesses the application and considers whether the child would benefit from a stay. Overall, children are accepted unless they do not fulfill the age group specifications, have severe medical symptoms (either psychiatric or physical) or are considered in need of a placement with more support than what can be offered at the camps e.g., for maladjusted children. The camp program is considered a pedagogical offer, not a psychiatric one. If the principal has any uncertainties of whether the child has medical issues, the application is sent to a pediatrician affiliated with DCSH. The pediatrician considers if the applicant could benefit from a stay based on the medical issues. E.g., children are accepted if they are diagnosed with only mild symptoms of a psychiatric disease e.g., ADHD. The principal can also arrange for an early meeting with the child and family to disclose whether the child is believed to benefit from a stay.

#### The Flow of Children at the Health Camps

**Figure 1:**
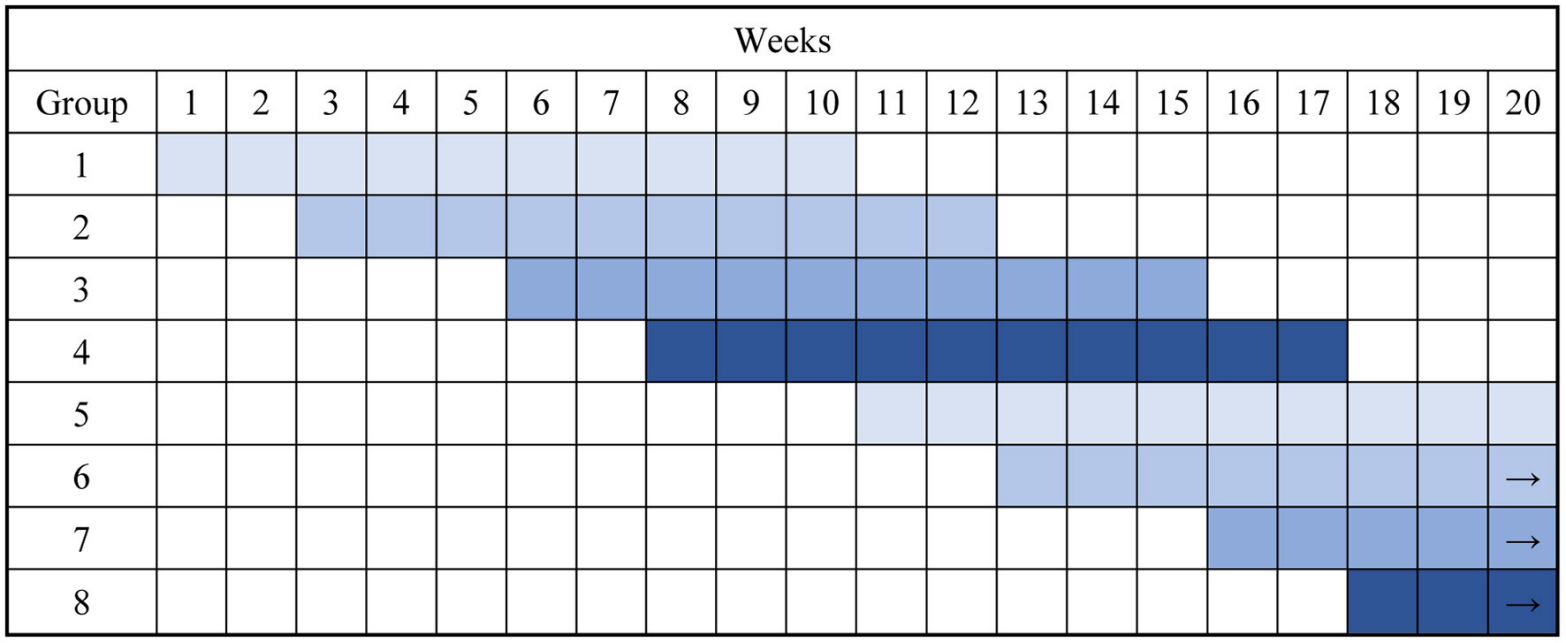
Example of the flow of children at the health camps with four groups at the time.

A group consists of 8–12 children depending on the capacity of each of the different camps, and there are three or four groups at each camp at the same time (Figure 1). The allocation to a group is based mainly on the child's placement on the waiting list which is determined by the reception data of the child's application. Considerations are given to provide balanced gender and age distributions within the groups.

The children do activities both within the groups and together with all participants at the camps. Each group has a team of pedagogues affiliated and each child is allocated a contact pedagogue from the team who takes care of any issues of concerns and communicates with their parents if needed. At the camp there is a continuous overlap between groups. One group completes their 10-week stay on a Friday; the next group starts the following Monday. The two to three other groups at the camp are ~2, 5 and 7 weeks into their stay. After 2–3 weeks, the drill repeats to a total of 10 weeks per group (Figure 1). The argument for doing this is that the health camps have experienced that the children who are already attending the camp can help the new children getting into the DSCH routines etc. Another advantage of this approach is that all children at some time point get the experience of being ‘the old ones' with experience and surplus of mental resources. They can support the new group of children, when they experience homesickness or other challenges from being away from home, which is common.

### Study Design

The effectiveness of the extended maintenance intervention will be evaluated in a quasi-experimental design (30) comparing the intervention group and the control group at long-term follow-up. Parallel to this we will conduct a process evaluation study.

#### Sampling and Allocation to Intervention Groups

Half of the children from the Liljeborg health camp is offered the extended maintenance intervention. The allocation to the intervention group is done by a secretary at the DCSH. The allocation is not random but based on the home municipality of the child (geographical criteria). The Liljeborg health camp covers both municipalities in the Capital Region of Denmark and the Region of Zealand and the distribution is ~50/50 from each region. All 10 municipalities in the Region of Zealand covered by the Liljeborg health camp are allocated to the intervention group. The control group includes children from the Liljeborg health camp along with the four other health camps receiving the standard maintenance intervention (control group 1) and children from the Liljeborg health camp allocated to the standard maintenance intervention (control group 2).

Blinding is not possible in this setting, and the standard maintenance intervention families at Liljeborg will have an idea of the activities going on in the extended maintenance intervention. For the four remaining health camps, families are not aware of any other type of maintenance interventions than the one offered at that specific home, which is the standard maintenance intervention.

#### Eligibility Criteria and Recruitment

##### Inclusion

All 7–14-year-olds who are signed up for a 10-week stay at one of the five health camps in the period from September 2019 to June 2021 will be enrolled in the effect evaluation. This also includes children who do not complete the health camp, according to the principle of intention to treat.

##### Exclusion

Some children will not be offered a maintenance intervention if they are only at the health camp for a few days. This is determined by the principal and the intervention coordinators at the five camps.

#### Control Group: Standard Maintenance Intervention

The standard maintenance intervention lasts ~9 weeks. It is initiated 5 weeks into the camp stay and continues 4 weeks after the stay. The standard maintenance intervention is delivered to the child and family by an intervention coordinator affiliated to each of the camps. The role of the intervention coordinator is to prepare and support the child upon return to the home environment. At two of the five camps this function is managed by a team pedagogue who is also present during the stay at camp and thereby knows the child well. At the three remaining camps the maintenance function is managed by a pedagogue that only gets to know the child sparsely during the stay but who receives inputs for the maintenance support from the pedagogues who know the child from the camp stay.

The support from the intervention coordinator consists of several activities of which the first is a conversation between the intervention coordinator and the family at the so called family day scheduled when the child has been at the camp for ~5 of the 10 weeks. On this day all parents can discuss current issues and get to know each other. The intervention coordinator gives a plenary presentation about potential pitfalls when the children return to their own home, meets each of the families for the first time, and has a short conversation with them, and produces an action plan in collaboration with each family. The action plan specifies what the family intends to do to help the child maintain the positive benefits of the stay, what changes should be made in the family, and how the parents will support these changes. The intervention coordinator guides the family in terms of who to address directly if help is needed from e.g., the municipality, a volunteer organization, or the school. The families are expected to make the contact to the school or the municipality themselves.

Within a few weeks after the first meeting and while the child is still at the camp, the intervention coordinator contacts the family by phone to follow up with support and advice on any issues regarding their action plan or any new concerns.

The family and the intervention coordinator meet again at the follow up day at the camp which is ~4 weeks after the child has returned to home. The children are often excited to see their friends, both the ones from the same group and the ones who are still at camp. For the parents there is room to discuss different issues and create a network among the parents. There is a presentation about what is often the most challenging issues when returning from health camp, good advice on healthy snacks and other relating issues. For most of the families this marks the end of the standard maintenance intervention period. For some families, the intervention coordinator might make some extra phone calls to the family if needed to follow up on specific issues.

#### Intervention Group: Extended Maintenance Intervention

The extended maintenance intervention is also delivered by an intervention coordinator who is assigned to a family. Some activities are identical to the standard maintenance intervention such as family day and follow up day along with phone calls (Table 1). The extended maintenance intervention encompasses several new initiatives compared to the standard maintenance intervention. It has a duration of ~16 weeks, 6 weeks while the child is at camp and 10 weeks afterwards. The intervention coordinator will participate in home visits and can also participate in different activities such as school visits and network meetings and assist the family in initiating new sports activities or other relevant activities (Table 2). The number of each of the activities are decided in corporation between the family and the intervention coordinator. The intervention has been in constant development since the beginning of 2018 and it is an ongoing process.

**Table 1:** Main similarities and differences between the two maintenance interventions.

**Family collaboration**	**Standard maintenance intervention (control group)**	**Extended maintenance intervention (intervention group)**
Intervention provider	Intervention coordinator	Intervention coordinator
Duration	9 weeks	16 weeks
Intervention concept	Standard	Tailored to each child
Information day before camp	X	X
Family day during camp	X	X
Follow up day 4 weeks after camp	X	X
Phone call/texts to follow up	X	X
Home visits during and after health camp		X
Network meetings		X
School meetings		X
Meeting with health worker or social worker in home municipality		X
Start-up of sports activities		X

**Table 2:** Activities in the extended maintenance intervention and frequency.

**Activities**	**Number**
**Home visits:** The intervention coordinator visits the child's home and talks to the parents and the child, most often separately. The first visit is while the child is still at camp and therefore solely involves a talk with the parents	2–5
**Network meetings:** The intervention coordinator arranges a meeting with relevant participants e.g., parents, schoolteacher, school principal, social and/or health worker from the municipality etc.	0–2
**School meeting:** The intervention coordinator visits the schools along with the child to tell the classmates about the child's experiences at the camp	0–1
**Meetings with health worker or social worker in home municipality**: The intervention coordinator can have contact to relevant people in the home municipality to insure relevant support to the family.	0–3
**Conversations with child:** If the intervention coordinator finds it necessary; she can have talks with the child alone. It is most common in the teenage children.	0–3
**Support to start up new sports activities:** The intervention coordinator supports the child in considering and identifying local sports offers and clubs to join. Sometimes the coordinator is presents at the child's first practice.	0–1
**Follow up by phone, e-mail, text messages:** The parents or child have contact with their intervention coordinator in between visits and activities as needed by the family.	0–??

The intervention coordinators' extended maintenance intervention in each family is guided by the so-called Life Satisfaction Compass, which is developed by the staff at the Liljeborg health camp and based on Prochaska and DiClemente's Stages of Change model (31). In collaboration with the staff at the Liljeborg health camp and the family's own intentions, the intervention coordinator uses the compass as a screening tool to identify the needs of each child and family to customize the intervention to them. It has eight focus areas: workout habits, dietary habits, network, family culture and structure, coping strategies, leisure life and communities, school life, and the child's general life satisfaction. Thus, the intervention may be tailored to address all or only a few areas of the compass important for the specific child and family. It is a dynamic intervention where the intervention coordinator continuously evaluates what needs to be the focus for the specific child or family. The intervention is designed to be easy and meaningful for the families to participate in since the intervention coordinators visit them at home and customize the intervention to each family making. Also, it is considered okay if the families do not feel like they need the help and therefore end the extended maintenance intervention, although it seems like very few families do this.

There are four intervention coordinators at the Liljeborg health camp with an educational background as pedagogues. They only get to know the children sparsely during the stay and they get their knowledge through meeting with the pedagogues working at the Liljeborg health camp. The extended maintenance intervention is organized in four settings identified in the program theory (see below), which are thought to influence how well a child adjusts to coming home after a stay at Liljeborg health camp and maintain his or her goals:

*1: The home setting:* The intervention coordinator visits the family and helps them with any concerns e.g., how to incorporate healthy habits in their everyday life, how to help their child with educational and social concerns at school e.g., friends and bullying. The intervention coordinator may take on many roles, but the main task is to help the family to a better understanding of their child's challenges and in many ways act as a discussion partner and present different tools to overcome challenges.

*2: The school setting*: The intervention coordinator sometimes helps the child in preparing a presentation for his or her school class about the time at the health camp and the developing journey, they have been through. The intervention coordinator sometimes also has a continuous dialogue with the schoolteachers to support the child, most often by email or phone.

Another activity is network meetings. During a network meeting, the family and the intervention coordinator meet with relevant professionals e.g., schoolteachers, principal at the home school, or caseworkers from the health or social department at the municipality to collaborate about the best ways to help the child. In some cases, a follow up meeting is arranged about 1 month later to pick up any challenges which have been faced in the period.

*3: The municipality setting:* The intervention in this setting depends on what challenges the child or family experiences. The intervention coordinator contacts the municipality and with her expertise and knowledge of the child, she tries to find a good way to help the child along with the municipality. If the main problem is overweight, then the child will be part of an offer for children with overweight in the municipality. If the problem is more of a social character, the caseworker at the municipality can help with different initiatives. Stakeholders from the municipality might also participate in network meetings as mentioned above.

*4: Leisure time setting:* This covers both if the child goes to an after-school center or any sports or other leisure time activities. In the after-school center the intervention coordinator can e.g., help with a dialogue about availability of heathy food and activities. The intervention coordinator can both help the family find a relevant leisure time activity or even help the child participate in the first session. It is preferred however, that the family find a leisure time activity themselves as it gives them ownership and thereby helps to maintain participation.

#### Developing a Program Theory of the Extended Maintenance Evaluation

The extended maintenance intervention is developed by the DCSH. It is based partly on recommendations from the previously mentioned evaluation from 2016 (27), recommending an extended collaboration with stakeholders in the home environment. However, the ideas for its content grew along the way and the actual development was done in collaboration between the principal and the intervention coordinators at Liljeborg health camp as an ongoing process. It is based on practical experiences, and with a focus on the narrative approach of changing the child's story telling about themselves to a more positive one as mentioned above. The ideas and assumptions behind the intervention were written down by one of the intervention coordinators during the development process, but in very general terms and there existed no explicit program theory when the research group entered the project. Thus, in order to plan a thorough process and effect evaluation design and avoid a black box evaluation we aimed to identify the exact content of the intervention and elicit the assumed but tacit working mechanisms of the intervention (32).

We started out by reviewing internal documents at the Liljeborg health camp concerning all five health camps. It consisted mainly of working manuals describing everyday life at the health camps, the activities and daily structure at the camps, the collaboration with families and the pedagogical philosophy determining the theoretical approach to the children as mentioned above. The documents also contained a description of the intervention content of the extended maintenance intervention at Liljeborg health camp, the allocation strategy of the maintenance intervention and a description of the self-invented ‘life satisfaction compass'.

**Figure 2:**
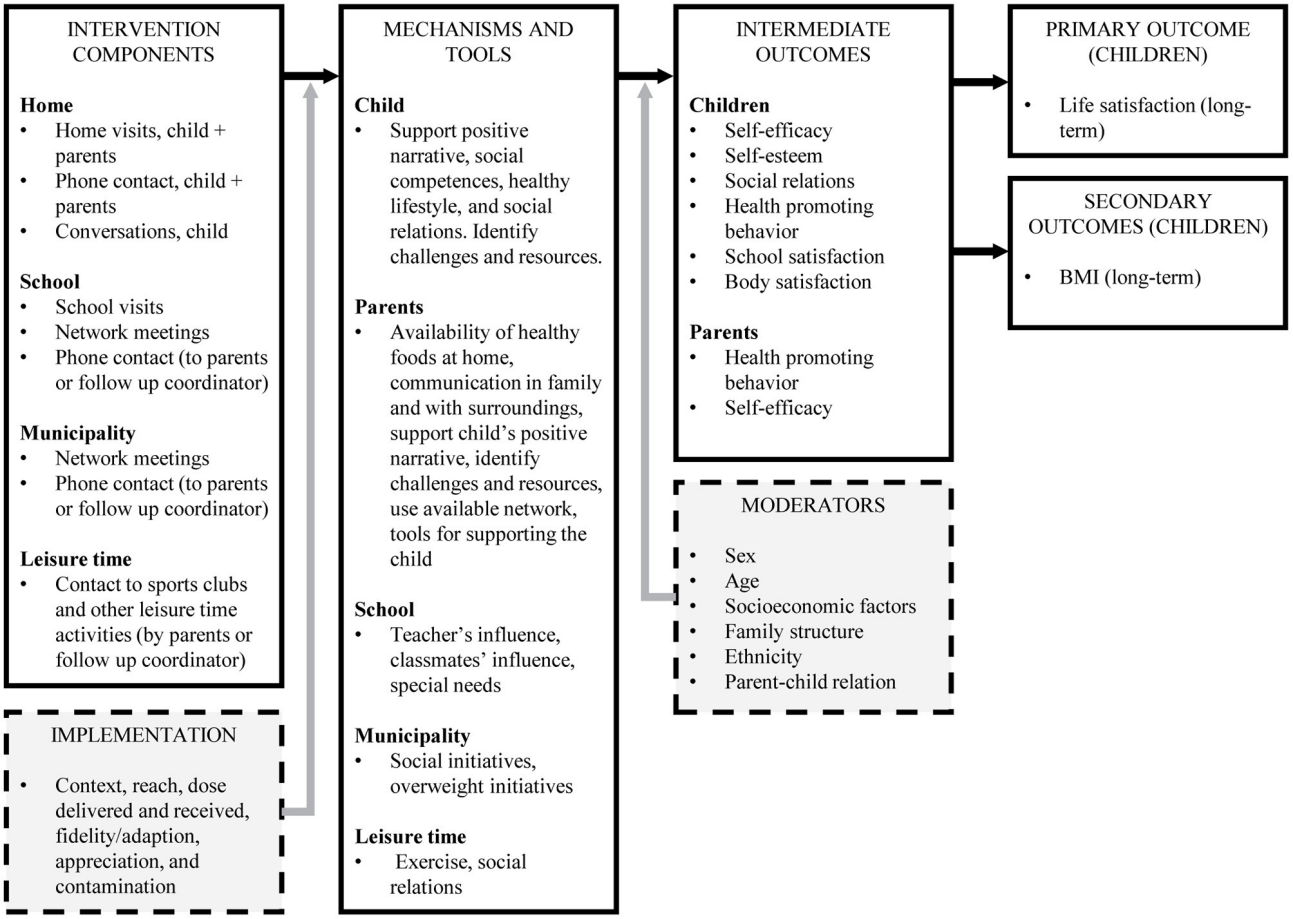
Program theory for extended maintenance intervention.

Secondly, we conducted a two-day program theory workshop with the four intervention coordinators, the principal from the Liljeborg health camp, and the director of DCSH along with the research group. The aim was to find out what had been the initial idea behind the extended maintenance intervention and how the activities were believed to influence the outcome. E.g., home visits were believed to make certain differences through specific mechanisms e.g., securing availability of healthy food and thereby influencing the intermediate health promoting behavior which was believed to help maintain a long-term effect on life satisfaction and BMI. See Figure 2 for the program theory.

The two program theory workshops showed that the extended maintenance intervention works at multiple settings: (1) home (children and parents/family), (2) school, (3) municipality, and (4) leisure activities. For each child and family, the intervention coordinators perform an individualized assessment as to what areas of the program theory the extended maintenance intervention needs to address to support the child's health and life satisfaction.

Through the program theory workshops, the intervention coordinators, and the principal from the Liljeborg health camp visualized how the extended maintenance intervention contains several elements anticipated to influence different aspects in the lives of the child and family.

BMI is defined as a secondary outcome in the program theory, even though it is not defined as such by the DCSH. Both the intervention at camp and the extended maintenance intervention have special focus on weight related behavior with healthy food, physical activity, and weight status at least two times during the camp.

### Data Collection

#### Data for Effect Evaluation

The data will be collected at the five health camps through questionnaires filled out by both children and parents. The web-based questionnaires are distributed by the DCSH as it is part of their routine data collection. Data are collected at the following time points: (1) Baseline prior to the 10-week health camp, (2) Post health camp on the last day of the health camp, (3) Short-term follow up 3 months after health camp, and (4) Long-term follow up 1 year after completion of camp (primary effect analysis). For both children and parents' questionnaires, existing questionnaires used by the DCSH were expanded by the research group. The parents will receive a reminder by email and eventually by phone if necessary. Data collection and reminders are managed by the DCSH.

##### Child Questionnaires

The questionnaires were expanded by the research group and new items were added by the research group. The items were based on scientific literature, experience from child surveys in general and existing questionnaires from the DCSH. The majority of items were taken from the Health Behavior in School-aged Children (HBSC) study (33). In order to identify items which reflects areas the children find especially important when returning to their homes, we also performed two focus group interviews among a total of 12 children at the Liljeborg health camp who had been home for 4 weeks. The final questionnaire was pilot tested in 24 children. The pilot test showed that the youngest children or children with reading difficulties had difficulties in reading some of the items. Therefore, we decided to add audio reading to the questionnaires which enables a child to have the items read aloud if needed.

The first two questionnaires will be filled out at the health camp which means that the response rate is expected to be close to 100% for these two questionnaires for the children who complete a stay at health camp. A schoolteacher or a pedagogue at the camp will be encouraged to help especially younger children as well as older children if they are unsure about the meaning of an item. The short-term and long-term questionnaires are web-based questionnaires which are sent to the parents' email addresses.

The questionnaires at baseline and long-term follow up are identical. In the post health camp questionnaire items about context at home e.g., school environment and friends are taken out since the children have not been part of everyday life in their home environment for 10 weeks. The short-term follow up questionnaire is identical to baseline and long-term questionnaires but with a few items about how it has been to return home from health camp and if they feel they got enough support from their surroundings.

##### Parent Questionnaires

The parent's questionnaires will be filled out by one or both parents or a guardian. The questionnaires are expanded from existing questionnaires from the DCSH and new items are developed from items modified from the HBSC to apply to parents, covering birth date and year of the child along with ethnic background and some items developed specifically for the study e.g., family structure. This was done to reduce the number of items in the children's questionnaire as this was already considerable large especially for the younger children. Furthermore, we include items covering socioeconomic factors from The National Representative Health and Morbidity Studies (SUSY) (34) along with items on lifestyle modified from the HBSC and DCSH so that both children and parents answer similar questions. We decided to add audio reading to the parent questionnaires as well as the DCSH argued that quite a few of the parents have reading difficulties.

#### Data for Process Evaluation

Process evaluation is recommended by the Medical Research Council in complex intervention (35). If the effect evaluation is done in isolation, it may leave essential questions unanswered and in the case of ineffectiveness we will not be able to differentiate between implementation or theory failure (35). Thus, the overall aim of the process evaluation study is to measure implementation of the extended maintenance intervention to enable analyses of how implementation fidelity affects the effect of the primary and secondary outcomes (per protocol analyses).

To detect levels of fidelity we will measure if the intervention coordinators implement the intervention to their initial goals as part of the action plan in order to identify possible implementation failures or to identify if the intervention and its assumptions for the working mechanisms suffer from theory failure.

In the case of effect, we want to be able to characterize if certain aspects of the intervention seem more likely to result in an effect and if e.g., if certain combinations of activities seem more effective.

Furthermore, we will identify facilitators and barriers for the implementation of the extended maintenance intervention to guide refinement of the intervention before it is up scaled to other DCSH health camps.

The process evaluation study will be structured according to the program theory and key process evaluations measured inspired by the Medical Research Council guidelines (35), a systematic process evaluation protocol (36), and the conceptual framework presented by Steckler AND Linnan (37). The process evaluation will combine multiple quantitative and qualitative data collection methods including questionnaires, workshops, participant observations, and interviews.

##### Children and Parent Questionnaires

Children and parents in the intervention group at Liljeborg health camp, will complete a short extra questionnaire on primarily process evaluation measures at the follow up reunion day at camp 4 weeks after the completion of the residential stay. Furthermore, a few items in the short-term follow up questionnaire relate to process evaluation measures. At both time points, we mainly will collect data on dose received by the children and families.

##### Intervention Coordinator Questionnaires

The intervention coordinators will be asked to complete three web-based questionnaires on process evaluation measures for each of the families in the intervention group at the following time points: (1) In the beginning of extended maintenance intervention ~4 weeks into the health camp, (2) In the middle of the extended maintenance intervention ~4 weeks after health camp and (3) At the end of the extended maintenance intervention ~10 weeks after the health camp.

Furthermore, to assess the actual content of the standard and extended maintenance intervention the intervention coordinators at Liljeborg health camp will complete an activity log for all the children in both intervention and control groups measuring amount of home visits, network meetings, phone calls etc.

Similarly, the intervention coordinators at the four control health camps will be asked to complete an activity log to assess similarities and differences in the activities delivered between intervention sites.

Due to the dynamic and versatile nature of the intervention, we will develop measures which are inspired by goal attainment scaling which measure progress in heterogeneous populations with a variety of treatment goals (38). Based on the Life Satisfaction Compass, we will ask intervention coordinators in the extended maintenance to complete questionnaires regarding planned focus areas in the compass before the intervention, focus areas during the intervention, and what the intervention actually ended up containing.

**Figure 3:**
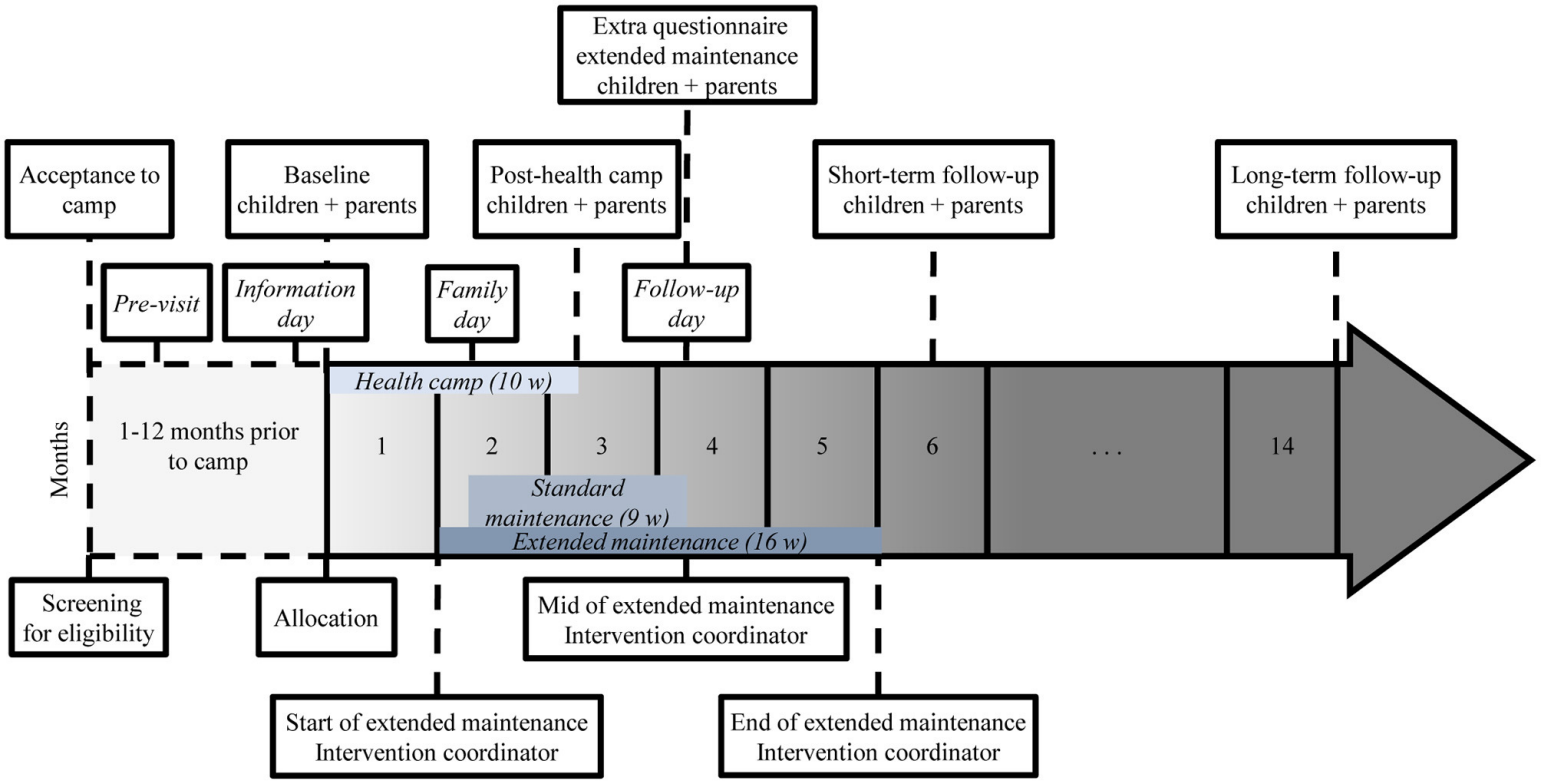
Participant timeline including time points for questionnaires.

See Figure 3 for participant timeline including timepoints for questionnaire distribution.

##### Preparation for the Qualitative Studies

As preparation for the process evaluation study, we held a two-day workshop at the Liljeborg health camp with the four intervention coordinators in the beginning of the project period to identify their perceptions of facilitators and barriers of their implementation of the intervention. These findings will guide the qualitative data collection for the process evaluation. We asked the intervention coordinators to list their thoughts of facilitators and barriers in each of the eight focus areas in the life satisfaction compass of the intervention. Inspired by thematic analysis (39), we rearranged all the facilitators and barriers into six new themes:

1. *The family's competences* or opportunities for supporting the health and life satisfaction of the child.
2. *The family's conditions* e.g., accommodation situation, diseases in the family, divorce, working hours, economy, which can all influence the family's ability to support health and life satisfaction of the child.
3. *Cooperation* around the child between parents, intervention coordinators and collaborators in the public sector such as in the school, municipality, and voluntary organizations.
4. *School culture* concerning school environment and management at the school.
5. *The child's skills, competences, and experiences* from the time before health camp and from the time at health camp e.g., self-esteem, self-efficacy and both positive and negative experiences in the social communities.
6. *The family's lifestyle* and the home environment e.g., availability of healthy food, physical activity level etc.

The workshop gave us a view of how the intervention coordinators experienced the overall facilitators and barriers of implementation of the intervention while the children's and families' experiences were missing. Consequently, we will focus the qualitative research on exploring children's, parents', and the intervention coordinators' views on the extended maintenance intervention with an essential focus on the families' competences and conditions along with a focus on the cooperation around the child.

We will specifically explore two cross-cutting facilitators which were highlighted by the intervention coordinators as having a great importance for the implementation of the intervention:

The establishment of a meaningful *relationship* between the intervention coordinator and families. The intervention coordinators found that this formed the basis of trust and was important for their opportunities for facilitating changes in the families and representing the families at e.g., network meetings with schoolteachers and other stakeholders.

1. *Parental self-efficacy and responsibility* in how to act and react in relation to their child's life satisfaction and healthy lifestyle.
2. We will also explore whether the families and intervention coordinators agree on the content of the intervention and if they think the intervention is a meaningful way to maintain high life satisfaction among children after the camp.

##### Qualitative Data Collection

*Observations*. We will conduct observations (40) in eight families in the intervention group. We will mainly participate in home visits and for two of the families also in network meetings with schoolteachers and other stakeholders with focus on the themes mentioned above.

*Interviews*. Interview guides will be inspired by the Theoretical Domains Framework (41). Firstly, we will carry out individual interviews (40) with each of the four intervention coordinators at Liljeborg health camp. The aim is to view the extended maintenance intervention from the perspective of an intervention coordinator covering how they meet a new family, what is on the agenda, how they document their work, and if they feel well equipped to implement the intervention. We will also interview the principal of the Liljeborg health camp as he is a key person in the development and refinement of the extended maintenance intervention.

Four of the eight observed families will be invited for interviews of both child, parent, and affiliated intervention coordinator. The aim of these interviews is to get a more thorough understanding of the different perspectives in the intervention with similarities and differences among the families and intervention coordinators.

##### Qualitative Data Analysis

The qualitative data will be analyzed by the principles of thematic analysis (39) and inspired by principles from collaborative data analysis (42). The collected qualitative data material will be read and discussed thoroughly by the whole research team. The analytic collaboration makes it possible to apply different analytic perspectives and interpretations of the data materials, thereby not one perspective dominates the analytic process.

### Outcomes

The outcomes of the study are summarized in Table 3. The questionnaires are mainly based on measures from the HBSC study (33) which are validated on 11–15 year-old children. They are supplemented with measures from the Danish National Youth Study (DNYS) (43) or already existing questions from DCSH. The items were either transferred directly or adjusted according to the present study population e.g., for bedtimes earlier time options were added. A few items were developed specifically for this study.

**Table 3:** Outcomes.

**Variable**	**Question**	**Item source**	**Data source**	**Time**	**Units/categories**
**Primary outcome**					
Self-reported life satisfaction	*Here is a picture of a ladder. Suppose the top of the ladder represents the best possible life for you and the bottom of the ladder the worst possible life. Where on the ladder do you feel you stand at the present time?*	Cantril ladder, HBSC	All children	– Baseline – Post health camp – Short-term follow up **– Long-term follow up**	0–10
**Secondary outcomes**					
Weight and height (BMI) Objective measures	*What is your weight (in kg)?*	HBSC	All children	Objective measures from the health camps at baseline and post health camp	Kg
	*What is your height without shoes (in cm)?*	HBSC	All children	Objective measures from the health camps at baseline and post health camp	Cm
Weight and height (BMI) Self-reported	*What is your weight (in kg)?*	HBSC	All children	– Baseline – Post health camp – Short-term follow up **– Long-term follow up**	Kg
	*What is your height without shoes (in cm)?*	HBSC	All children	– Baseline – Post health camp – Short-term follow up **– Long-term follow up**	Cm
**Explorative outcomes**					
Parent's report of child's life satisfaction	*Here is a picture of a ladder. Suppose the top of the ladder represents the best possible life for your child and the bottom of the ladder the worst possible life for your child. Where on the ladder do you feel he/she stands at the present time?*	Cantril ladder (modified to parents from HBSC)	Parents	– Baseline – Post health camp – Short-term follow up – Long-term follow up	0–10
Well-being	*Over the last two weeks: I have felt cheerful and in good spirits I have felt calm and relaxed I have felt active and vigorous I woke up feeling fresh and rested My daily life has been filled with things that interest me*	WHO-5(52)	All children	– Baseline – Post health camp – Short-term follow up – Long-term follow up	5 (all the time) to 0 (at no time)
	*In the last 6 months: how often have you had the following…? headache, abdominal pain, backache, feeling low, irritability or bad temper, feeling nervous, difficulties in getting to sleep or feeling dizzy*	HBSC Symptoms checklist	All children	– Baseline – Post health camp – Short-term follow up – Long-term follow up	4 (almost every day) to 0 (never)
**Intermediate outcomes**					
Self-efficacy	*How often can you find a solution to problems if you try hard enough?*	HBSC	All children	– Baseline – Post health camp – Short-term follow up – Long-term follow up	5 (always) to 0 (never)
	*How often can you manage the things you decide to do?*	HBSC	All children	– Baseline – Post health camp – Short-term follow up – Long-term follow up	5 (always) to 0 (never)
	*How often are you able to handle unexpected situations?*	Modified from ‘Projekt Optur'(53)	All children	– Baseline – Post health camp – Short-term follow up – Long-term follow up	5 (always) to 0 (never)
Self-esteem	*I like myself*	HBSC	Children 10–14 years	– Baseline – Post health camp – Short-term follow up – Long-term follow up	4 (strongly agree) to 0 (strongly disagree)
	*I am good enough as I am*	HBSC	Children 10–14 years	– Baseline – Post health camp – Short-term follow up – Long-term follow up	4 (strongly agree) to 0 (strongly disagree)
	*Others my age like me*	HBSC	Children 10–14 years	– Baseline – Post health camp – Short-term follow up – Long-term follow up	4 (strongly agree) to 0 (strongly disagree)
Social relations	*How many days a week are you normally with friends in your leisure time outside school or after school care?*	Modified from HBSC	Children 10–14 years	– Baseline – Post health camp – Short-term follow up – Long-term follow up	7 (never) to 0 (7 days a week)
	*How often are you in contact with friend over the internet or on your phone?*	HBSC Modified	Children 10–14 years	– Baseline – Post health camp – Short-term follow up – Long-term follow up	5 (Don't know) to 0 (Almost all the time every day)
	*How often have you been bullied at school in the past couple of months?*	HBSC	All children	– Baseline – Post health camp – Short-term follow up – Long-term follow up	4 (several times a week) to 0 (I have not been bullied at school in the past couple of months)
	*In the past couple of months how often have you been cyberbullied?*	HBSC	Children 10–14 years	– Baseline – Post health camp – Short-term follow up – Long-term follow up	4 (several times a week) to 0 (I have not been cyberbullied in the past couple of months)
	*How easy is it for you to talk to the following persons about things that really bother you?*	HBSC Added answer-categories	All children	– Baseline – Post health camp – Short-term follow up – Long-term follow up	4 (very easy) to 0 (Don't have or see this person)
	*I express my opinion when I think something is unfair*.	HBSC	Children 10–14 years	– Baseline – Post health camp – Short-term follow up – Long-term follow up	3 (almost always) to 0 (almost never)
	*I try to understand my friends when they are sad or angry*.	HBSC	Children 10–14 years	– Baseline – Post health camp – Short-term follow up – Long-term follow up	3 (almost always) to 0 (almost never)
	*I am good at working with others in a group*	HBSC	Children 10–14 years	– Baseline – Post health camp – Short-term follow up – Long-term follow up	3 (almost always) to 0 (almost never)
	*I ask my friends for help when I am in trouble*.	HBSC	Children 10–14 years	– Baseline – Post health camp – Short-term follow up – Long-term follow up	3 (almost always) to 0 (almost never)
Health promoting behavior	*How many times a week do you usually eat or drink…?* • *Fruit* • *Vegetables* • *Candy/chocolate/ chips/ice cream/cake* • *Coke/soda with sugar* • *Fast food (e.g. pizza, kebab, shawarma, burgers, or sausages)*	Combination of HBSC and DCHS	All children	– Baseline – Post health camp – Short-term follow up – Long-term follow up	6 (never) to 0 (every day, more than once)
	*How many times a week do you eat…* • *Healthy breakfast* • *Healthy lunch* • *Dinner with your family*	Combination of HBSC and DCSH	All children	– Baseline – Post health camp – Short-term follow up – Long-term follow up	5 (never) to 0 (every day)
	*When do you normally fall asleep on a school night?*	Developed for study	Children 10–14 years	– Baseline – Post health camp – Short-term follow up – Long-term follow up	8 (20.00 or earlier) to 0 (24.00 or later)
	*When do you usually go to bed if you have to go to school the next morning?*	HBSC Added answer-categories	All children	– Baseline – Post health camp – Short-term follow up – Long-term follow up	8 (20.00 or earlier) to 0 (24.00 or later) Added categories compared to HBSC
	*When do you usually wake up on school mornings?*	HBSC Changed answer-categories	All children	– Baseline – Post health camp – Short-term follow up – Long-term follow up	6 (5.00 or earlier) to 0 (8.00 or later)
	*How many hours a week do you usually exercise so much that you lose your breath or sweat in school or outside school?*	Modified from HBSC	All children	– Baseline – Post health camp – Short-term follow up – Long-term follow up	5 (none) to 0 (7 hours a week or more)
School satisfaction	*Are you happy about your school?*	DCSH	All children	– Baseline – Post health camp – Short-term follow up – Long-term follow up	5 (Yes, always) to 0 (No, never)
	*Do you feel part of the community in your class?*	The Well-being Despite Study(54)	All children	– Baseline – Post health camp – Short-term follow up – Long-term follow up	4 (there is no community in my class) to 0 (always)
	*Most students in my class(es) are kind and helpful*	HBSC	Children 10–14 years	– Baseline – Post health camp – Short-term follow up – Long-term follow up	4 (strongly agree) to 0 (strongly disagree)
	*Other students accept me as I am*	HBSC	Children 10–14 years	– Baseline – Post health camp – Short-term follow up – Long-term follow up	4 (strongly agree) to 0 (strongly disagree)
Body satisfaction	*Are you satisfied with your body?*	Danish Youth Profile(43)	All children	– Baseline – Post health camp – Short-term follow up – Long-term follow up	9 (10 very satisfied) to 0 (1 very dissatisfied)
Parental health promoting behavior	*State how often your family* • *Eat candy, chips, ice cream, cake, or chocolate* • *Eat fast food (e.g. Pizza, kebab, shawarma, burgers, or sausages)* • *Eat fruit* • *Eat vegetables* • *Are observant of portion size* • *Drink coke or soda with sugar*	Modified from HBSC and DCSH	Parents	– Baseline – Post health camp – Short-term follow up – Long-term follow up	6 (never) to 0 (every day, more than once)
	*State how often your family* • *Eat foods marked with whole-grain symbol* • *Eat food with ‘healthier choice' symbol*	Modified from HBSC and DCSH	Parents	– Baseline – Post health camp – Short-term follow up – Long-term follow up	7 (never) to 0 (do not know the symbol)
	*State how often your family* • *Eat breakfast together* • *Eat dinner together*	Modified from HBSC and DCSH	Parents	– Baseline – Post health camp – Short-term follow up – Long-term follow up	5 (never) to 0 (every day)
	*How often do you usually exercise in your free time so much that you get out of breath or sweat?*	Modified from HBSC	Parents	– Baseline – Post health camp – Short-term follow up – Long-term follow up	5 (every day) to 5 (rarely or never)
	*How often does your partner usually exercise in his or her free time so much that he or she get out of breath or sweat?*	Developed for study	Parents	– Baseline – Post health camp – Short-term follow up – Long-term follow up	6 (every day) to 0 (I do not have a partner)
Parental self-efficacy	*How often can you find a solution to your child's problems if you try hard enough?*	Modified from HBSC	Parents	– Baseline – Post health camp – Short-term follow up – Long-term follow up	4 (always) to 0 (never)
	*How often can you handle an unexpected situation your child gets into?*	Modified from ‘Projekt Optur'	Parents	– Baseline – Post health camp – Short-term follow up – Long-term follow up	4 (always) to 0 (never)
**Process evaluation measures**					
Implemen-tation measures	*Context, reach, dose delivered and received, fidelity/adaption, appreciation, and contamination*	Developed for study	Children + parents	– Baseline – Extra questionnaire – Short-term follow up	
	*Context, reach, dose delivered and received, fidelity/adaption, appreciation, and contamination*	Developed for study	Intervention coordinators	Start, mid, end intervention questionnaires	

The primary outcome is children's life satisfaction 1 year after completion of camp (long-term follow up), and will be measured in the child questionnaire by an adapted version of the Cantril ladder used in the HBSC study questionnaire (44). The outcome will be measured at all four time points.

The secondary outcome is self-reported BMI 1 year after completion of camp (long-term follow up). It will be measured objectively twice at the five health camps by the personnel on their scale at the beginning and end of the 10-week health camp (baseline, post health camp) and self-reported by the child in the questionnaires at all four time points.

### Sample Size Calculations

**Figure 4:**
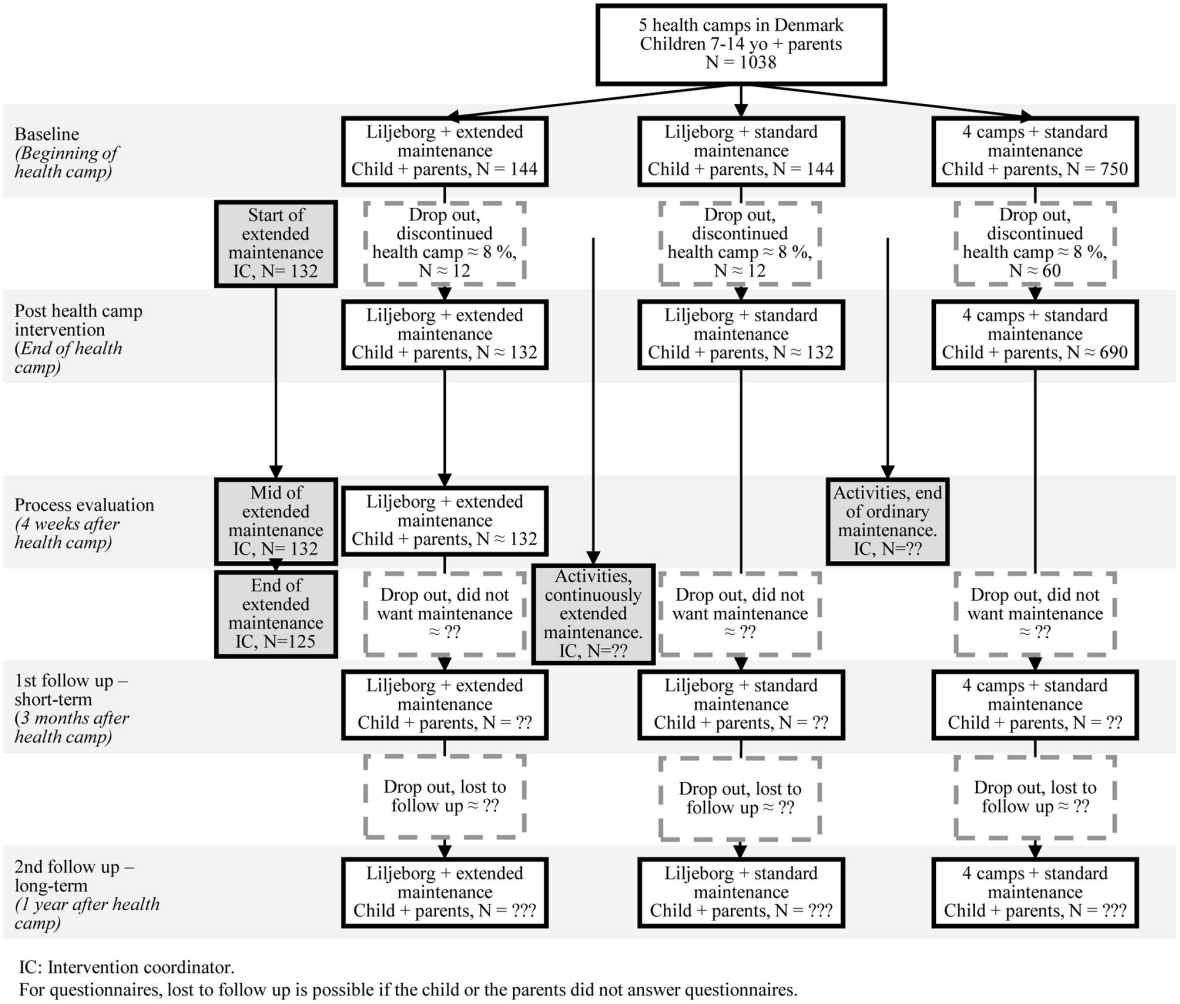
Flowchart of expected numbers of participants.

In total 1,038 children will attend the 10-week stay at one of the five camps during the recruitment period (see Figure 4). Approximately 288 will attend Liljeborg health camp and half (~144) will receive the extended maintenance intervention and the other half (~144) the standard maintenance intervention. The 750 children from the four other residential health camps are offered a standard maintenance intervention as well. We know from records at the health camps, that approximately 8% do not complete their stay at the camps. We do, however, have no knowledge of how many children turn down the offer of standard or extended maintenance.

Historically it has been difficult to reach a satisfactory response rate for the long-term follow up questionnaires as mentioned before (27).

We used already collected questionnaire data from the camps to calculate the number of children needed to detect changes on the primary outcome (see Table 3). We made a random retrieval of 50 children from each of the five health camps, a total of 250.

Based on this calculation, we found a SD of 2.1. A prospective power calculation shows that with 80% power, we will be able detect a difference of 0.53 points on the Cantril ladder between groups with 144 in the intervention group and 894 in the control group. These power calculations are based on the assumptions that all the children complete the stay at the camp and all the children allocated to the extended maintenance intervention at the Liljeborg health camp, will accept this. If we assume there is a 8% drop out of all health camps, the calculation with 132 in the intervention group and 822 in the control group, we will be able to detect a difference of 0.55 on the Cantril ladder, making it a very small difference.

### Data Analysis

We will use descriptive statistics to investigate baseline equivalence and loss to follow up. We will graphically visualize the development over time in both intervention group and control group 1 for the primary and secondary outcomes.

We will analyze the effectiveness of the extended maintenance intervention compared to control group 1 on the primary and secondary outcomes, using linear regression analysis and on explorative outcomes using logistic regression analyses. We will apply an intention-to-treat approach including all children in the intervention group they initially have been allocated to irrespective of whether they have completed the intervention or are lost to follow-up. Missing data will be replaced by multiple imputation. We will perform complete case analysis for sensitivity analyses.

The effect analyses will be adjusted for baseline factors including gender and age of the child, life satisfaction, BMI-*Z* score, and parental socioeconomic position.

For the total population at all five camps, we will calculate the standard deviation of the primary and secondary outcomes right after camp to calculate Cohen's *d* effect size.

Furthermore, we will perform sensitivity analysis restricted to the data from the Liljeborg health camp to identify any differences between camps (control group 2). We will investigate the association between intervention dose and effect (per protocol analyzes).

We will investigate if the effect differs by subgroups (see moderators in Figure 2).

We will summarize process measures using appropriate descriptive statistics including proportions, means (SD), and medians (IQR).

We will identify who is in most need of the intervention and find out who does not need the intervention.

### Protocol Amendment

All amendments to the protocol will be reported in the trial registration and be transparent in study papers.

We have already been obliged to introduce some amendments to the original trial registration due to the Covid-19 pandemic and modifications made by the intervention coordinators along the way. We originally planned to recruit children for a 52-week period but due to the fact that the Liljeborg camp initiated the online questionnaires faster than the other four health camps, we have extended the recruitment at Liljeborg camp with 10 weeks to obtain a larger intervention group. Furthermore, the covid-19 pandemic has influenced the recruitment period as the health camps have been locked down by the government twice during the year of recruitment along with shorter local lock downs due to outbreaks of covid-19 and periods of lower occupancy rates at the camps e.g., due to requirements from the government to have less people gathered in settings like this.

We originally planned to include objective measures of weight and height at baseline, post health camp and at the long-term follow-up at the Liljeborg health camp. However, due to the Covid-19 lockdown DCSH will not be able to gather all children at the Liljeborg health camp to participate in a reunion day 1 year after camp and obtain long-term objective measures of weight and height as originally planned. Instead, we must rely on self-reported measurements at all timepoints supplemented by findings on agreement between objective and self-reported measurements at baseline and post camp.

## Discussion

This paper describes the study protocol for a quasi-experiment of an extended maintenance intervention, which aims to maintain positive benefits of an initial 10-week stay at a residential health camp on children's life satisfaction and BMI after they have returned to their homes. Both the initial intervention at the health camp, the standard maintenance intervention (control group) and the extended maintenance intervention are developed by practitioners and delivered for other purposes than research.

Center for Intervention Research, at The National Institute of Public Health, SDU in Denmark was commissioned by the Liljeborg Foundation and the DCSH to conduct an external research-based evaluation of the implementation and effectiveness of the intervention. It is however challenging to evaluate real world practice in a rigorous research design.

The practical set-up of the health camps and the maintenance interventions limited our methodological opportunities and forced us to make some compromises to the ideal research design. We would have preferred to conduct a RCT study, but it will not be possible to randomize children into intervention and control groups as the DSCH controls the allocation procedure. Instead we will approach the evaluation as a quasi-experiment with the risk of selection bias (30). We do not expect large systematic differences between the two groups as the DSCH's allocation procedure is motivated by which municipality the child comes from and both intervention and control groups recruit children from municipalities with a different socioeconomic range. However, we will assess baseline equivalence between the two samples using descriptive statistics on key variables and we will adjust the effect analyses for key covariates chosen a priori. The study is also limited by the self-reported height and weight measures at 3 months and 1 year follow up (45, 46). However, we hypothesize that a potential disagreement between objective measures and self-reported measures will be similar in both intervention and control groups. The objective and self-reported measures at baseline and post camp will enable us to explore this hypothesis.

The strengths of this study include the use of external evaluators, the systematic approach to evaluation planning, the research-based evaluation design, the prospective trial registration, and the development of a program theory to structure the evaluation.

There was no explicit program theory for the extended maintenance intervention when we entered the study. We developed a program theory together with intervention providers to structure the evaluation and prevent ‘black box' evaluation (32). As external evaluators it was highly important that we spent time on getting to know the organization and on reaching a common understanding of the purpose and working mechanism of the intervention with intervention providers (47). During the development of the program theory, it became apparent how each of the intervention coordinators and the principal had their own tacit assumptions about the purpose and working mechanism of the intervention. Thus, the workshop on program theory also benefitted the organization as they were made aware of their different views on working mechanism (48).

### Perspectives

It is an ongoing challenge to maintain effects of public health interventions in the long-term e.g., positive changes in life satisfaction or weight loss. Additionally, long-term effects of interventions are often not measured nor reported. This quasi-experimental study will provide new knowledge on the potentials and effectiveness of individualized maintenance interventions on long-term effects on life satisfaction and weight loss among children. The qualitative process evaluation will provide knowledge on what kind of support families need to be able to help their child maintain a healthier lifestyle and the physical, mental, and social health benefits from the health camp as they return to their home environment.

Practicians and researchers call for practice-based research in order to produce findings which are relevant to practicians and may refine and improve current practice (49). It is however challenging to evaluate real world practice in a research design. Real world practice is often developed and implemented based on tacit assumptions, experiential knowledge, ‘practice wisdom'(50) and rules of thumb rather than theory, evidence, and by use of systematic planning tools. We present a systematic approach to evaluate real world practice in a strong research design along with the related challenges and potentials. This may inspire other researchers who are commissioned to evaluate real world practice.

Levels of implementation and fidelity are positively associated with intervention effects (51, 52). It is however challenging to assess the implementation level in individualized, tailored, dynamic interventions such as the extended maintenance intervention where the exact content, dosage and length of the intervention for each child and family is based on the “clinical” judgement of the intervention provider. Our study will propose new approaches to assess the fidelity and the level of implementation in such interventions inspired by goal attainment scaling (38).

## Ethics and Dissemination

### Ethical Issues

In spite of good intentions, public health interventions may have unintended consequences at both the organizational and individual level (53). We will explore potential unintended consequences of the extended maintenance interventions as part of the qualitative and quantitative studies. The extended maintenance intervention is presumed to have a positive effect on body satisfaction as the children become healthier, lose weight, and get higher self-esteem. However, the focus on health and weight loss at camp could potentially result in body dissatisfaction (54). We will investigate the direction of this association as part of the effect analyses on explorative outcomes.

Another ethical challenge is the fact, that we have been informed that the evaluation will have implications for the future of the extended maintenance intervention. If the evaluation does not show an effect it is uncertain if and in what form the intervention will continue and therefore also whether the intervention coordinators can keep their jobs. Thus, our presence as evaluators might put them in an uncomfortable situation where they feel like they are being judged on their job abilities. We will pay attention to this especially during our observations and interviews and highlight that the aim of the qualitative study is to understand the perspectives of the intervention of the participants and providers, the working mechanisms of the intervention along with facilitators and barriers for implementation. Our task is not to evaluate whether they are doing their job sufficiently. Furthermore, we will not evaluate the effectiveness of the intervention based on the qualitative study. The effect study is based on child questionnaires. To address some of these uncertainties, it is important for us as evaluator to have a good understanding of the intervention confer the workshops on program theory and barriers and facilitators and to talk openly with the intervention coordinators about any concerns they might have concerning the evaluation study. Furthermore, we will pay attention to not exposing any of the intervention coordinators in the dissemination of the qualitative findings.

### Dissemination

The trial results will be communicated to other researchers through peer-reviewed journals and scientific conferences. We will disseminate the results to the public including the DCSH and municipalities using press releases, scientific reports, lay summaries, and oral presentations.

## Ethics Statement

Ethical review and approval was not required for the study on human participants in accordance with the local legislation and institutional requirements. Written informed consent from the participants' legal guardian/next of kin was not required to participate in this study in accordance with the national legislation and the institutional requirements.

## Author Contributions

MK is project manager of the study and participated in developing the design of the evaluation, prepared questionnaires for the quantitative data collection and will participate in the qualitative data collection. She will head the data cleaning, data analysis and interpretation of data. MK wrote the first draft of this manuscript and completed the final version of the manuscript. RK and SM participated in developing the design of the evaluation, the preparation of questionnaires and will contribute to the data analysis, and interpretation of data. MR participated in developing the design of the evaluation and the preparation of questionnaires. PD participated in the design of the evaluation and preparation of questionnaires. LT advised on the planned statistical analyses and will participate in the data analysis and interpretation of data. All authors read, revised, and approved the final version of the manuscript.

## Funding

The PhD-scholarship is sponsored by Liljeborg Fonden (the Liljeborg Foundation) (info@liljeborgfonden.dk) and University of Southern Denmark (SDU). The Liljeborg Foundation requested an external evaluation and neither the Liljeborg Foundation nor University of Southern Denmark have been or will be involved in the study design of the evaluation, data analysis, or interpretation of data.

## Conflict of Interest

PD is now on the board of the Liljeborg Foundation. However, she was not on the board when the grant was given or when she participated in the development of the evaluation design or of the questionnaires as she took office in May 2020. Furthermore, she will not participate in the data analysis or interpretation. The remaining authors declare that the research was conducted in the absence of any commercial or financial relationships that could be construed as a potential conflict of interest.

## Publisher's Note

All claims expressed in this article are solely those of the authors and do not necessarily represent those of their affiliated organizations, or those of the publisher, the editors and the reviewers. Any product that may be evaluated in this article, or claim that may be made by its manufacturer, is not guaranteed or endorsed by the publisher.

## Abbreviations

DCSH: Danish Christmas Seal Houses
BMI: Body Mass Index
BMI *Z*-score: Standardized body mass index score for specific populations
HBSC: Health Behavior in School-aged Children
WHO: World Health Organization
IC: Intervention coordinator
SD: Standard deviation
IQR: Interquartile range
SDU: University of Southern Denmark
RCT: Randomized controlled trial
NVK: The Danish National Committee on Health Research Ethics
RIO: Research and Innovation Organization.
